# Impact of High-Pressure Processing on Prevention of Quality Loss and Spoilage Bacteria Diversity in Precooked Baby Clam (*Paphia undulata*) During Refrigerated Storage

**DOI:** 10.3390/foods14081421

**Published:** 2025-04-20

**Authors:** Suriya Palamae, Umesh Patil, Pitima Sinlapapanya, Hui Hong, Yadong Zhao, Bin Zhang, Soottawat Benjakul

**Affiliations:** 1 International Center of Excellence in Seafood Science and Innovation, Faculty of Agro-Industry, Prince of Songkla University, Hat Yai 90110, Thailand; suriya.pal@psu.ac.th (S.P.); umesh.p@psu.ac.th (U.P.); pitimasinlapapanya@gmail.com (P.S.); 2 Beijing Laboratory for Food Quality and Safety, College of Food Science and Nutritional Engineering, China Agricultural University, Beijing 100083, China; hhong@cau.edu.cn; 3 Key Laboratory of Health Risk Factors for Seafood of Zhejiang Province, College of Food Science and Pharmacy, Zhejiang Ocean University, Zhoushan 316022, China; zhaoyd@zjou.edu.cn (Y.Z.); zhangbin_ouc@163.com (B.Z.); 4 Department of Food and Nutrition, Kyung Hee University, Seoul 02447, Republic of Korea

**Keywords:** bacterial community, aquatic product, food spoilage, non-thermal processing, eating quality

## Abstract

The influence of high-pressure processing (HPP) at 200, 400, and 600 MPa on spoilage bacterial diversity, microbial load, and chemical and sensory properties of the precooked edible portion of baby clam (PC-EP) was investigated. HPP at 400 MPa for 4 min (400 MPa) significantly prolonged shelf life and sensory acceptability up to 12 days, maintaining a total viable count (TVC) below 6 log CFU/g. In contrast, the TVC of both the control (without HPP treatment) and 200 MPa-treated samples exceeded this limit by day 0 and 3, respectively. The 400 MPa-treated samples showed a reduced load of psychrophilic bacteria, *Aeromonas* species, and lactic acid bacteria over 12 days. Additionally, coincidentally lower total volatile base and trimethylamine levels confirmed the good quality of HPP-treated PC-EP. Based on next-generation sequencing, a significantly lower microbial diversity index was found in the 400 MPa-treated samples, and it was dominated by *Carnobacterium*, *Lactococcus*, and *Psychrobacter* on day 12. In contrast, the control harbored spoilage bacteria, including *Lactococcus*, *Aeromonas*, *Shewanella,* and *Pseudomonas*, which correlated with higher acetic acid and acetoin levels as confirmed by HS-SPME-GC-EI/MS. These findings demonstrated that HPP at 400 MPa for 4 min was an effective non-thermal preservation method, extending the shelf life of PC-EP by inhibiting spoilage bacteria.

## 1. Introduction

Baby clam, or undulated surf clam (*Paphia undulata*), is a commercially valuable bivalve that inhabits shallow marine environments as a benthic species [1]. It is widely distributed along Thailand’s southern coast, Southeast Asia, and Southern China. In 2022, Thailand produced 2.6 thousand tonnes of baby clams with a value of THB 175.4 million [2]. By 2023, production had upsurged to 8.2 thousand tonnes [2], all of which was derived from capture fisheries. Baby clams are highly valued due to their unique flavor, nutritional benefits (low fat with high protein), and medicinal properties [3]. However, their high perishability and short shelf life associated with high water activity (Aw), nutrient content, and neutral pH pose significant problems for fishermen and the seafood industry.

Keeping baby clam at low temperature is common practice, which can lengthen the shelf life by 2–3 days [4]. Although vacuum packing combined with low-temperature storage is widely adopted owing to its efficiency, low cost, and technological maturity, the rapid microbial growth related to quality loss is still an obstacle. Precooked baby clam edible portion (PC-EP), packed and stored under iced or refrigerated conditions, is frequently sold in markets or supermarkets across Thailand as the primary method for preserving baby clams. In addition, whole fresh baby clams are another form available in the fresh market with a very short shelf life. Heat-based shucking methods using hot water or steam effectively soften the adductor muscle, favoring the separation of the edible portion (EP) from the shells [5]. This process also helps inactivate certain microflora in the bivalve EP [6]. However, the diversity of spoilage bacteria and chemical changes that occur during refrigerated storage of PC-EP are not fully understood. Additionally, undesirable aromas, off-flavors, and off-odors caused by microbial decomposition, lipid oxidation, and enzymatic reactions make the product unsuitable for sale and consumption [7].

Microbial growth, particularly that of specific spoilage organisms (SSOs), is a key factor in the deterioration of stored seafood. SSOs are fast-growing microbes with high spoilage potential, and their proportion increases as the storage time extends. The most common SSOs in aquatic products are Gram-negative bacteria, including *Aeromonas*, *Lactococcus*, *Shewanella*, *Pseudomonas*, etc. These bacteria accelerate spoilage, leading to quality loss and consumer rejection of aquatic products. *Aeromonas*, for instance, is a prominent spoilage bacterium known for its ability to degrade proteins and lipids, especially under refrigeration conditions, making it a major contributor to cold-chain deterioration. Furthermore, *Aeromonas* has the ability to form biofilms, complicating its elimination and producing thermostable proteases and lipases that generate off-odors, leading to rapid spoilage. Lactic acid bacteria (LAB) produce sour and off-flavors through the formation of organic acid, and their protein degradation can result in bitterness [8].

In recent years, interest in minimally processed seafood has grown continuously due to its ability to retain nutritional value and fresh-like qualities. Among emerging technologies, high-pressure processing (HPP) has gained recognition as an effective method for eliminating spoilage and pathogenic microorganisms. HPP has been demonstrated to inactivate *Vibrio* species, aerobic bacteria, H_2_S-producing bacteria, and psychrotrophic bacteria in various fresh shellfish, including Asian hard clams (*Meretrix lusoria*) [9], oysters (*Crassostrea gigas*) [10], blood clams (*Tegillarca granosa*) [11], and razor clams (*Sinonovacula constricta*) [12]. However, the effects of HPP on the preservation and shelf life extension of PC-EP during refrigerated storage have not been elucidated. Additionally, there is a lack of comprehensive studies regarding the impact of HPP on spoilage bacteria diversity and chemical and sensorial changes of PC-EP during refrigerated storage.

This study aimed to assess the effectiveness of HPP at varying pressure levels (200, 400, and 600 MPa) for 4 min on the microbiological, chemical, and sensorial quality of refrigerated PC-EP. Additionally, spoilage bacterial communities and volatile organic compounds of PC-EP during refrigerated storage were also investigated.

## 2. Materials and Methods

### 2.1. Chemicals and Microbial Media

All chemicals were acquired from Sigma-Aldrich (St. Louis, MO, USA), while microbial media were purchased from Oxoid (Thermo Fisher Scientific, Waltham, MA, USA).

### 2.2. Treatment of Precooked Baby Clam Edible Portion with and Without HPP

PC-EP from Klamai Sealand Company Ltd., Mueang Surat Thani, Surat Thani province, Thailand, was used in this study. PC-EP stored at 4 °C was obtained from a supermarket in Hat Yai, Thailand, during November–December 2024. The PC-EP had an average weight of 4.5 ± 0.5 g and an average length of 1.3 ± 0.4 cm. The company’s shucking process for PC involved boiling whole fresh baby clams (100 °C, 5 min), followed by immediate immersion in iced water to terminate further cooking and facilitate shell removal. The PC-EP (500 g) was packed in a plastic bag, transported in ice, and displayed in a supermarket at 4 °C within 48 h before being purchased.

The PC-EP obtained from the supermarket was then vacuum-packed using a packaging bag (130 × 200 mm^2^, 35 µm thickness) and sealed under vacuum for 15 sec with a vacuum packing machine (Model Audionvac VM203, Audiovac, Weesp, the Netherlands). Basically, for each run, 2 bags (500 g/bag) were purchased, pooled, and used as the composite samples. Thereafter, 8 clams were randomly collected and placed in each bag and vacuum-sealed. Each vacuum-sealed bag containing PC-EP, as described above, was placed in a high-pressure processing (HPP) machine (Model HPP600 MPa/5 L, Jiujiu, Baotou KeFa High Pressure Technology Co., Ltd., Baotou City, Inner Mongolia Autonomous Region, China) [11]. Water, the pressure-transmitting medium, was transferred into the 5 L chamber. The pressure was gradually increased from 0.1 MPa to the target levels (200, 400, or 600 MPa) within 1.5 min. The HPP treatment was maintained for 4 min and subsequently released within 10–15 s. Throughout the process, the chamber temperature was controlled at 5 ± 2 °C. Three bags were treated with HPP at each level of pressure used. The average values for each parameter tested were recorded. Two different runs were carried out in the same manner. The samples subjected to HPP for 4 min were designated as HPP-treated groups, while the unpressurized samples (0.1 MPa, vacuum-sealed) served as controls. During storage for 15 days at 4 ± 2 °C, the samples were analyzed at 3-day intervals.

### 2.3. Physical Analyses

After HPP treatment under different pressure levels, the surface appearance and expansion rate were evaluated [11]. Photos of the control PC-EP and its pressurized counterparts were recorded using ImageJ software (Version 1.47a, National Institute of Health, Bethesda, MD, USA). Surface area (cm^2^) and expansion rate (%) were reported. Other analyses of PC-EP were also carried out.

### 2.4. Chemical Analyses

#### 2.4.1. pH Value

For pH measurement, 5 g of PC-EP samples were homogenized with 20 mL of deionized water (10,000 rpm, 1 min) using a homogenizer (model: IKA Labortechnik, Selangor, Malaysia). The pH was then determined using a pH meter (model: pH 700, Eulech Instrument, Singapore).

#### 2.4.2. Protein Patterns

Protein patterns were analyzed using sodium dodecyl sulfate–polyacrylamide gel electrophoresis (SDS-PAGE) under both reducing and non-reducing conditions [13]. For sample preparation, 3 g of the sample was mixed with 27 mL of 5% (*w*/*v*) sodium dodecyl sulfate solution, heated at 85 °C for 60 min, and then centrifuged at 8000× *g* for 10 min. Protein concentration was measured using the Biuret method, and 30 µg of protein was loaded onto a freshly prepared polyacrylamide gel (12% resolving gel and 4% stacking gel) prior to electrophoresis. Protein separation was conducted using a Mini Protein II unit (Bio-Rad, Hercules, CA, USA). After electrophoresis, the gel was stained with Coomassie Brilliant Blue, followed by destaining to visualize the protein bands [11].

#### 2.4.3. Total Volatile Basic-Nitrogen (TVB-N) and Trimethylamine-Nitrogen (TMA-N) Contents

TVB-N and TMA-N contents were determined [14]. The same procedure was used for both assays, except that formaldehyde solution was mixed with the extract to fix primary and secondary amines for analysis of TMA-N content, as tailored by Prabhakar et al. [14].

#### 2.4.4. Volatile Compounds

Volatile compounds were analyzed using HS-SPME-GC-EI/MS with an Agilent 7890B GC-7000D MS system (Santa Clara, CA, USA). A 75 µm Carboxen/PDMS SPME fiber was used for extraction. Identification was performed by comparing spectra against the Wiley 10, NIST14, and NIST17 libraries, ensuring a match score above 90%. Each compound was reported as abundance [15].

### 2.5. Microbiological Analysis

At each sampling time, three separate bags (*n* = 3) were taken and analyzed [15]. The plate count method was used to assess viable bacterial counts. The total viable count (TVC) was determined using plate count agar (PCA, code: CM0463). Lactic acid bacteria (LAB) were quantified on de Man Rogosa Sharpe (MRS) agar (code: CM0333B) under anaerobic conditions. Presumptive *Aeromonas* spp. were identified using *Aeromonas* agar base (code: CM0833) supplemented with ampicillin selective supplement (code: SR0136). The psychrophilic bacteria count (PBC) was determined using PCA as the medium, in which the plates were incubated at 4 ± 2 °C for 1 week. For other microbial counts, the incubation was done at 35 ± 2 °C for 24 h. The microbial colony counts were expressed as log CFU/g.

### 2.6. Next-Generation Sequencing

Bacterial populations were examined using 16S rRNA gene next-generation sequencing (NGS) [16]. PC-EPs from 0.1 MPa (day 0), HPP-treated at 400 MPa (day 0), as well as 0.1 MPa and 400 MPa samples after 12 days of refrigerated storage were analyzed. DNA was first extracted using the ZymoBIOMICS^®^-96 MagBead DNA Kit (Zymo Research, Irvine, CA, USA), following the manufacturer’s guidelines. The V3-V4 regions of the 16S rRNA gene were amplified through polymerase chain reaction (PCR).

Sequencing was performed using the Illumina^®^ MiSeq^TM^ platform with a v3 reagent kit (600 cycles) and a 10% PhiX spike-in to enhance sequencing accuracy. Bioinformatics analysis was done using Uclust from QIIME v.1.9.1 for taxonomic classification. Composition visualization was performed with the aid of the Zymo Research Database.

### 2.7. Sensory Evaluation

Only samples with TVC below the acceptable limit (6 log CFU/g) were employed for assessment. The selected PC-EP samples were boiled for 10 min before being tested. A panel of 50 individuals (37 males and 13 females, aged 25–40 years) assessed the samples using a 9-point hedonic scale, as described by Morten et al. [17]. A score below 5 indicated unacceptability of the tested samples [18].

### 2.8. Experimental Design and Statistical Analyses

A completely randomized design (CRD) was used for all experiments. Sensory evaluation was conducted using a randomized complete block design (RCBD). All tests were done in triplicate, and the statistical analysis involved one-way and two-way ANOVA. A mean comparison was carried out using Tukey’s test.

Operational taxonomic unit (OTU) analysis was conducted for taxonomic study using Usearch 7, and clustering was performed via Uparse 7.0.1090. Alpha diversity analysis was carried out using Mothur 1.30.2. All data were processed through the ZymoBIOMICS^®^ Service (Zymo Research, Irvine, CA, USA).

## 3. Results and Discussion

### 3.1. Appearance of Precooked Baby Clam Edible Portion Subjected to HPP at Various Pressure Levels

**Figure 1:**
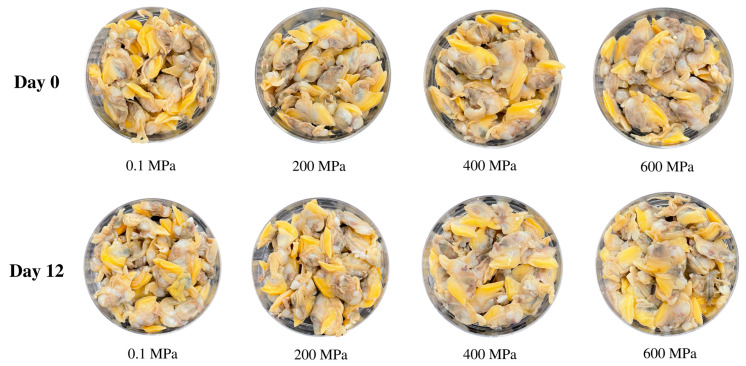
Photographs of the edible portions of precooked baby clams without and with HPP at 200, 400, and 600 MPa for 4 min on day 0 and day 12 of refrigerated storage.

The appearance of precooked baby clam (PC) edible portion (EP) or PC-EP subjected to HPP at various levels for 4 min is shown in Figure 1. The control sample (no HPP treatment) had a light yellow and opaque appearance with an irregular shape. When HPP was applied, particularly at pressures above 400 MPa, the samples became glossier. At 600 MPa, the PC-EPs appeared slightly larger in size than the control and other HPP-treated samples. HPP treatment significantly influenced the surface area and expansion rate of PC-EP (*p* < 0.05). As pressure increased, both parameters showed a progressive rise. For the control, the surface area was 3.66 cm^2^ (considered 100%). When pressure upsurged to 200 MPa and 400 MPa, the surface area expanded to 3.86 cm^2^ (106.2%) and 4.04 cm^2^ (118.8%), respectively. The highest expansion rate was observed at 600 MPa, with a surface area of 4.12 cm^2^ (121.3%). During HPP at high pressure, some bonds were destroyed, making the EP looser in structure. When the release of pressure was done, the fluid from the PC-ER moved back into the HPP-treated sample at higher levels. This was witnessed by the upsurged surface area with a coincident increase in expansion. No marked differences between the same sample on day 0 and day 12 of refrigerated storage (Figure 1).

### 3.2. Protein Patterns of Precooked Baby Clam Edible Portion Subjected to HPP at Various Pressure Levels

**Figure 2:**
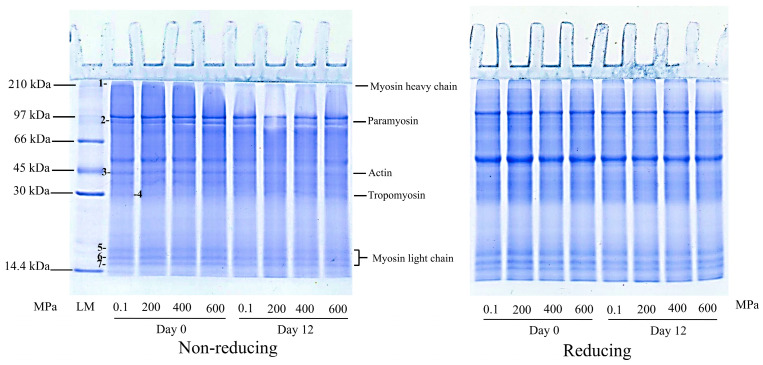
SDS-PAGE patterns of the edible portions of precooked baby clams without and with HPP at 200, 400, and 600 MPa for 4 min on day 0 and day 12 of refrigerated storage. LM: Low molecular weight marker.

**Figure 3:**
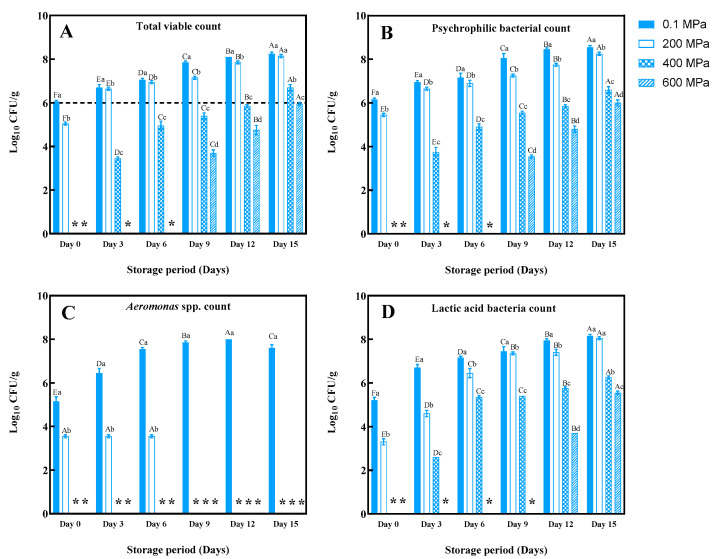
Change in total viable count (**A**), psychrophilic bacteria (**B**), *Aeromonas* spp. (**C**), and lactic acid bacteria count (**D**) in the edible portions of precooked baby clams without and with HPP at 200, 400, and 600 MPa for 4 min during refrigerated storage. Bars represent the standard deviation (*n* = 3). The dashed line represents 6 log CFU/g of total viable count, the regulatory standard limit. (*) represents no detection. Each symbol represent each sample. For example, ** indicates that each from both samples had no detectable microorganism. Different lowercase superscripts within the same storage time tested indicate significant differences (*p* < 0.05). Different uppercase superscripts within the same sample indicate significant differences (*p* < 0.05).

The control sample showed seven main protein bands having molecular weights (MW) of 17–210 kDa under both reducing and non-reducing conditions (Figure 2). Under reducing condition, some high-MW protein bands disappeared, indicating the presence of disulfide bonds. The identified major proteins included myosin heavy chain (MHC, 210 kDa, band 1), paramyosin (95 kDa, band 2), actin (43 kDa, band 3), tropomyosin (35 kDa, band 4), and myosin light chain (MLC, 17–21 kDa, bands 5–7). On day 0, the protein patterns of untreated and HPP-treated PC-EP at different pressure levels were similar, indicating that HPP had no immediate effect on the protein profile. However, after 12 days of refrigerated storage, a marked decrease in the band intensity of large-MW protein bands, especially paramyosin (95 kDa), was observed. Additionally, the intensity of the actin (43 kDa) and MLC (17–21 kDa) bands decreased over the storage period. The results indicated that muscle proteins were degraded, mainly by the contaminated bacteria, during processing and packing. As illustrated in Figure 3, the loads of spoilage organisms, such as *Aeromonas* and lactic acid bacteria, increased by day 12, aligning with the observed decrease in muscle protein band intensity, indicating their role in protein hydrolysis during storage. Lactic acid bacteria, *Pseudomonas* and *Shewanella* have been reported to produce extracellular proteases, in which these bacteria can use free amino acids as nutrients [19]. The degradation of muscle protein could be associated with the loss in textural properties, e.g., softened texture, as well as the formation of metabolite with undesirable odor or flavor [20,21].

### 3.3. Chemical Quality of Precooked Baby Clam Edible Portion Subjected to HPP at Various Pressure Levels

TVB-N and TMA-N contents serve as key indicators of seafood spoilage related to shelf life [14]. On day 0, TVB-N levels ranged from 0.79 to 1.11 mg N/100 g (Table 1). Among all samples tested, the control had the highest content (*p* < 0.05). Volatile basic compounds are primarily produced by spoilage microorganisms, leading to the accumulation of volatile bases, such as trimethylamine, dimethylamine, ammonia, and other compounds responsible for off-flavors in seafood [22]. According to Okpala [23], TVB-N levels categorize seafood quality as follows: <12 mg N/100 g (fresh), 12–20 mg N/100 g (slightly decomposed but edible), 20–25 mg N/100 g (borderline), and >25 mg N/100 g (inedible and decomposed). The initial low TVB-N levels indicated that the PC-EP samples were of high quality. TVB-N content upsurged over time in all samples, while the control sample consistently exhibited the highest levels throughout the storage period (*p* < 0.05). The content in this sample rose from 1.11 mg N/100 g on day 0 to 13.06 mg N/100 g at the end of storage. Among the HPP-treated samples, the 200 MPa-treated sample exhibited higher TVB-N levels than those subjected to HPP at ≥400 MPa on day 3 (*p* < 0.05), while similar values were noted between 400 MPa and 600 MPa (*p* > 0.05). On days 12 and 15, TVB-N levels in the 400–600 MPa-treated samples remained below the permissible limit (30 mg N/100 g), demonstrating the effectiveness of HPP in preserving PC-EP quality. In previous studies, chilling, modified atmosphere packaging (MAP), and sous vide processing have shown varying effects on TVB-N levels in seafood. For instance, chilled storage of precooked baby clams in air packaging typically results in a rapid increase in TVB-N, in which the levels approach or exceed the spoilage thresholds within 12 days at 4 °C [24]. MAP can delay spoilage, depending on gas composition and storage conditions. TVB-N levels in precooked baby clams remained below 30 mg N/100 g for up to 27 days [24]. Sous vide-processed mussels demonstrated greater stability, with TVB-N levels remaining within acceptable limits throughout 50 days of storage at 4 °C [25].

The TMA-N content, another indicator of seafood spoilage, varied between 0.25 and 0.33 mg N/100 g on day 0 and upsurged steadily over storage time (Table 1). Similar to the TVB-N results, the control sample showed the most pronounced increase in TMA-N levels throughout storage. By day 3, this sample showed the greatest TMA-N content (0.78 mg N/100 g), while TMA-N content of HPP-treated samples ranged from 0.40 to 0.57 mg N/100 g (*p* < 0.05). On day 6, the control sample possessed the highest content (1.13 mg N/100 g) (*p* < 0.05). The production of TMA-N is associated with the activity of spoilage bacteria such as *S. putrefaciens*, *P. phosphoreum*, *Aeromonas* spp., *Enterobacteriaceae*, and *Vibrio* spp., which convert trimethylamine oxide (TMAO) into TMA as induced by TMAO reductase, leading to undesirable odors and flavors [26]. On day 12, samples subjected to HPP at 400 and 600 MPa had the lowest TMA-N levels (*p* < 0.05), aligning well with a reduced microbial load. In contrast, the TMA-N content of the control sample reached 5.46 mg N/100 g, exceeding the acceptable seafood limit of 5 mg N/100 g [27]. This indicated spoilage in the untreated PC-EP. These findings confirmed that HPP effectively controlled the formation of TVB and TMA, thereby preserving PC-EP quality.

pH is another key indicator of seafood quality because it is linked to chemical reactions related to spoilage or chemical deterioration. The pH of the PC-EP samples was influenced by HPP, as shown in Table 1. Over the storage period, all samples exhibited a gradual pH decline, likely due to microbial activity, particularly LAB, which produces acidic metabolites under vacuum conditions. During 0–15 days, the pH of the 400 and 600 MPa-treated samples remained relatively stable. In contrast, the control and 200 MPa-treated samples exhibited a significant pH decrease (5.39–6.20) from days 9 to 15. Bivalves contain a high carbohydrate content, particularly glycogen. Glycogen undergoes bacterial fermentation, producing lactic acid and causing pH reduction [28]. This decline serves as a spoilage indicator for oysters and other shellfish, particularly those packaged under vacuum or micro-aerobic conditions [29]. In a bivalve quality assessment, a pH above 6.0 indicates “good” quality, while a pH below 5.0 signifies spoilage [9]. These findings highlighted the ability of HPP to maintain the pH of PC-EP during extended storage.

### 3.4. Microbiological Quality

The changes in microbiological loads for the control and HPP-treated samples during refrigerated storage are presented in Figure 3. The initial TVC, PBC, *Aeromonas* spp., and LAB counts for the control sample were 6.1, 6.2, 5.2, and 5.3 log CFU/g, respectively. Following HPP treatment at 400 MPa for 4 min, no viable microorganisms were detected for up to 3 days (Figure 3). In general, TVC, PBC, *Aeromonas* spp., and LAB counts remained lower at all storage times when the sample was treated with HPP at 600 MPa (Figure 3A–D). HPP at 400 and 600 MPa for 4 min effectively eradicated the bacteria. The intense pressure disrupts cell structures, leading to leakage of intracellular substances like ATP and causing cell death [30]. HPP-induced damage includes alterations in membrane permeability, protein denaturation, and genetic modifications. These alterations contributed to bacterial inactivation [31,32]. Based on the International Committee of Microbiological Specializations of Food (ICMSF), the acceptable limit for TVC in aquatic products is 6 log CFU/g [33]. In filter feeders like baby clam, TVC reflects microbial quality and hygiene during processing as well as the high microbial load in the habitat of bivalves, in which the contamination could take place in PC-EP with ease [34]. TVC above 6 log CFU/g presents a potential health risk.

On day 0, TVC across all samples ranged from undetectable to 6.1 log CFU/g (Figure 3A). The high TVC, especially in the control, reflected the huge load of microorganisms, which contaminated the PC-EP during handling, processing, and packing. In addition, the delay in the process might allow the contaminated bacteria to proliferate rapidly. In the control, the TVC increased continuously during storage. Conversely, the HPP-treated samples exhibited lower initial TVC. Seafoods, especially bivalves, are perishable due to their high water activity and nutrient content [35]. In addition, based on the feeding behavior, the microorganisms could be accumulated in the EP of bivalves with ease. The TVC for the control and 200 MPa, 400 MPa, and 600 MPa-treated samples reached 6.1 (day 0), 6.6 (day 3), 6.7 (day 3), 6.7 (day 15), and 6.0 log CFU/g (day 15), respectively, exceeding the acceptable limit. Therefore, HPP, especially at high pressure, could inactivate or harm bacteria contaminated in PC-EP, thus extending lag and stationary phases [36].

The PBC varied from undetectable to 6.2 log CFU/g on day 0 (Figure 3B). The highest PBC was found in the control, similar to that found for the TVC on day 0. On day 3, the PBC increased in all samples except for 600 MPa-treated ones. The control sample exhibited the highest PBC during storage (6.2–8.6 log CFU/g, from day 0 to 15). Higher pressure (≥400 MPa) effectively delayed PBC growth, compared to the control and 200 MPa-treated samples, demonstrating HPP’s effectiveness in mitigating spoilage caused by psychrophilic bacteria. HPP at 600 MPa showed the highest efficacy in inactivating the psychrophilic bacteria, especially during the refrigerated condition used for storage. HPP was reported to lower the growth of psychrophilic bacteria of blood clam (*Tegillarca granosa*) during refrigerated storage [33]. In addition, Lin et al. [9] reported that HPP could inactivate bacteria dominant in hard clam (*Meretrix lusoria*) samples during 9 days of refrigerated storage.

*Aeromonas* spp. showed an initial count of 5.3 log CFU/g in the control sample (*p* < 0.05) (Figure 3C). Over storage time, its count increased in the control and 200 MPa-treated samples (*p* < 0.05). However, no *Aeromonas* spp. were detected in the 400 MPa and 600 MPa-treated samples throughout storage, indicating high susceptibility of this bacteria toward HPP at high pressure levels. By day 9, *Aeromonas* spp. count in 200 MPa-treated samples declined to undetectable levels, likely due to refrigerated conditions, whereas the counts in the control samples continued to increase (*p* < 0.05).

The LAB count in the control and HPP-treated PC-EP samples (200–600 MPa) stored at 4 °C for 15 days is presented in Figure 3D. The highest initial count was observed in the control sample (5.2 log CFU/g, *p* < 0.05). LAB increased during storage across all samples. The highest levels were found in the control, and the lowest count was obtained in 600 MPa-treated samples on day 15 (*p* < 0.05). LAB causes seafood spoilage by producing sour and off-odors through organic acid formation [8]. Additionally, protein degradation by these bacteria imparts a bitter taste. LAB counts were higher in vacuum- and CO_2_-enriched modified atmosphere packaging [33,37], favoring their growth due to their anaerobic–aerotolerant nature. On day 12, a higher LAB count was found across all samples, ranging from 3.7 to 8.0 log CFU/g. However, HPP ≥ 400 MPa for 4 min effectively reduced LAB counts, compared to lower-pressure treatments (*p* < 0.05).

During cold storage, samples treated with HPP ≥ 400 MPa showed the impeded increases in TVC, PBC, *Aeromonas* spp., and LAB counts, compared to those subjected to lower-pressure treatments. Notably, HPP treatment prevented *Aeromonas* spp. recovery throughout storage, extending the shelf life of PC-EP to 12 days, while the control sample had a high load of all bacteria. However, HPP, especially at high pressure levels, could eradicate those bacteria to a high extent.

Overall, HPP at 400 and 600 MPa extended the shelf life up to 12 days, in which TVC was lower than the limit. Due to the lower energy required for the former, HPP at 400 MPa for 4 min was selected as the optimal condition for the treatment of PC-EP. In a previous study, Vongsawasdi et al. [24] demonstrated that MAP significantly extended the shelf life of precooked baby clams with an initial TVC of less than 2 log CFU/g. Using a gas mixture of 60% CO_2_, 20% O_2_, and 20% N_2_, clams stored at 4 ± 2 °C remained acceptable for up to 24 days, compared to 10 days for the sample stored under atmospheric packaging. CO_2_ acts as an antimicrobial through carbonic acid formation, while reduced O_2_ limits aerobic spoilage bacteria. Therefore, prior HPP in combination with MAP more likely offered better preservation than using HPP alone.

### 3.5. Bacterial Diversity of Precooked Baby Clam Edible Portion Subjected to HPP Under Selected Conditions

**Figure 4:**
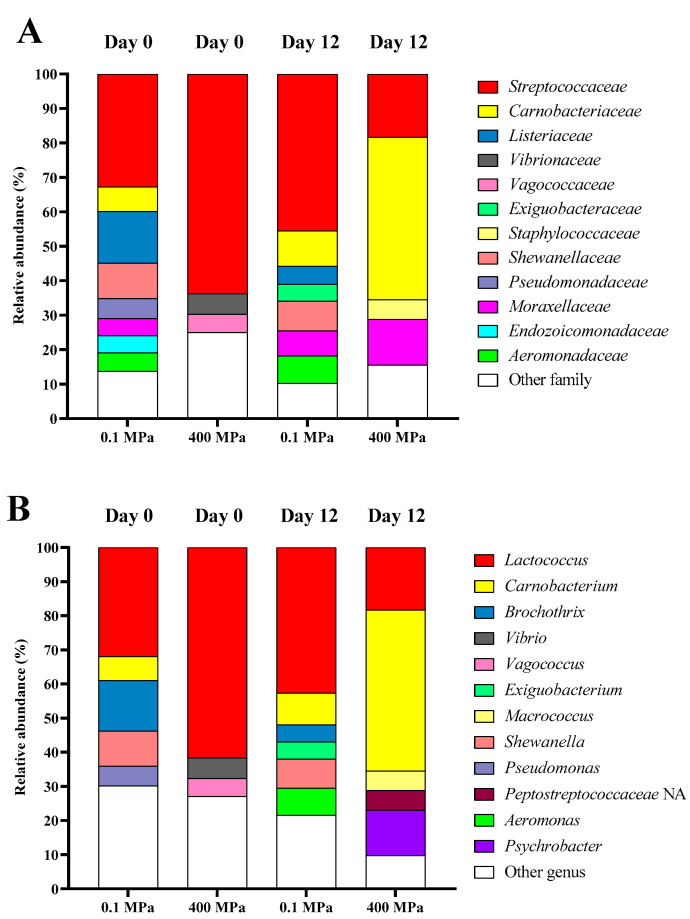
Relative abundance (%) of the taxonomies in the edible portions of precooked baby clams without and with HPP treatment at 400 MPa for 4 min on day 0 and day 12 of refrigerated storage. Data are presented at (**A**) the family level and (**B**) the genus level. Low-abundance species (<5%) and unassigned taxa were grouped under “Others”.

The microbial composition of PC-EP samples, including the control and HPP-treated samples, was analyzed at both the family and genus levels, as shown in Figure 4A,B. On day 0, the control sample exhibited a high relative abundance of *Streptococcaceae*, *Listeriaceae*, *Shewanellaceae*, *Carnobacteriaceae*, *Pseudomonadaceae*, *Aeromonadaceae*, *Moraxellaceae*, and *Endozoicomonadaceae*. On day 12, microbial diversity shifted slightly, in which *Streptococcaceae*, *Carnobacteriaceae*, *Shewanellaceae*, *Aeromonadaceae*, *Moraxellaceae*, *Listeriaceae*, and *Exiguobacteraceae* still remained dominant in the control sample, indicating microbial proliferation in the absence of HPP treatment. In contrast, the 400 MPa-treated sample initially contained *Streptococcaceae*, *Vibrionaceae*, and *Vagococcaceae* on day 0. On day 12, its microbial composition was significantly changed. *Carnobacteriaceae*, *Streptococcaceae*, *Moraxellaceae*, and *Staphylococcaceae* became prevalent. The notable reduction in *Shewanellaceae*, *Pseudomonadaceae*, *Aeromonadaceae*, and *Streptococcaceae* suggested that HPP effectively suppressed several bacterial populations, especially spoilage bacteria, during refrigerated storage. 

At the genus level, the control sample (day 0) was dominated by *Lactococcus*, *Brochothrix*, *Shewanella*, *Carnobacterium*, and *Pseudomonas*. On day 12, *Lactococcus*, *Carnobacterium*, *Shewanella*, *Aeromonas*, *Brochothrix*, and *Exiguobacterium* became predominant. These findings aligned with previous studies showing that *Shewanella*, *Aeromonas,* and *Pseudomonas* were the most dominant spoilage bacteria in seafoods, e.g., fish and shellfish [38]. For the 400 MPa-treated sample, *Lactococcus*, *Vibrio*, and *Vagococcus* were dominant on day 0. On day 12, spoilage-associated genera were declined, while *Carnobacterium*, *Lactococcus*, *Psychrobacter*, *Peptostreptococcaceae* NA, and *Macrococcus* were more prevalent. This shift in the microbial community highlighted HPP’s effectiveness in reducing spoilage and pathogenic bacteria during refrigerated storage.

**Figure 5:**
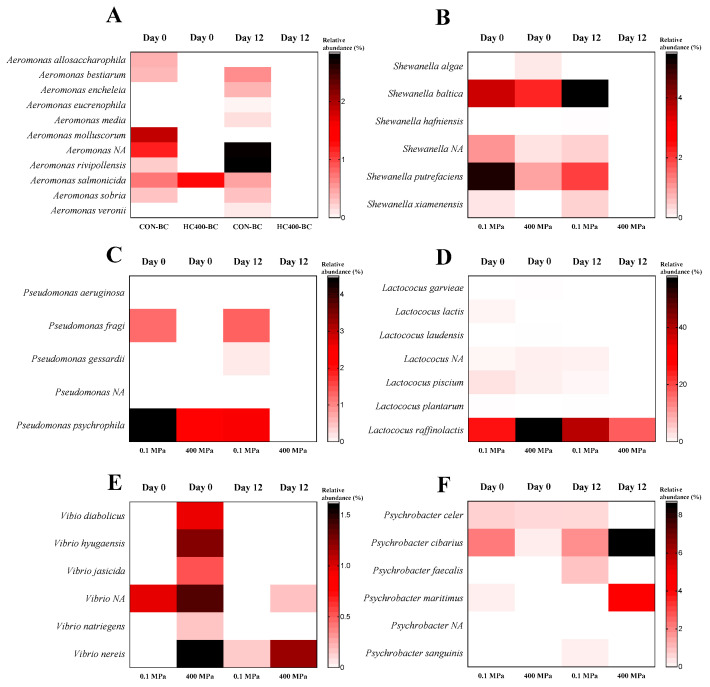
Heat map of the taxonomies at the species level in the edible portions of precooked baby clams without and with HPP treatment at 400 MPa for 4 min on day 0 and day 12 of refrigerated storage. Data are presented for *Aeromonas* species (**A**), *Shewanella* species (**B**), *Pseudomonas* species (**C**), *Lactococcus* species (**D**), *Vibrio* species (**E**), and *Psychrobacter* species (**F**).

A heat map analysis further confirmed significant microbial shifts between the untreated (control) and HPP-treated samples during refrigerated storage (Figure 5). After 12 days, HPP effectively controlled *Aeromonas*, *Shewanella*, and *Pseudomonas*, thus increasing the shelf life and enhancing microbiological safety (Figure 5A–C). *A. salmonicida* initially exhibited slight resistance to HPP at 400 MPa on day 0, but totally disappeared on day 12. Meanwhile, *A. sobria* and *A. bestiarum* were completely eliminated under HPP. In contrast, *A. rivipollensis* increased in the control samples, thereby supporting HPP’s ability to inhibit its proliferation. *S. putrefaciens* was significantly affected by HPP, decreasing from 5.2% in the control to 1.02% in the 400 MPa-treated sample on day 0 and becoming undetectable on day 12. Similarly, S. baltica declined from 3.63% to 2.31% after HPP treatment and was entirely inhibited on day 12. These results confirmed HPP’s effectiveness in controlling *Shewanella*, a key spoilage organism in seafoods. For *Pseudomonas*, *Ps. psychrophila* was the most prevalent species in the control, decreasing from 4.5% on day 0 to 2.6% on day 12. However, for the 400 MPa-treated sample, its levels dropped to 2.2% on day 0 and were undetectable on day 12. Additionally, *Ps. aeruginosa*, *Ps. gessardii*, and *Ps. fragi* were completely inactivated by HPP. This coincided with previous studies indicating that HPP effectively reduced psychrotrophic bacteria, which contribute to seafood spoilage. HPP also influenced the *Lactococcus* populations (Figure 5D). *L. raffinolactis* rose from 26.34% (day 0) to 40.13% (day 12) for the control. However, HPP and refrigerated storage significantly reduced its abundance, and the 400 MPa-treated sample had decreased to 18.31% by day 12. Additionally, *L. lactis* and *L. piscium* were eliminated by HPP on day 12. The presence of the *Vibrio* species was also significantly reduced by HPP (Figure 5E). While *V. diabolicus*, *V. hyugaensis*, and *V. jasicida* were detectable when treated with HPP at 400 MPa on day 0, they were absent on day 12. *V. natriegens* and *V. nereis* persisted at lower levels, further demonstrating HPP’s efficacy in controlling *Vibrio*, some of which are pathogenic. Among the *Psychrobacter* species, *P. cibarius* increased from 0.32 on day 0 to 8.73 on day 12 when HPP at 400 MPa was applied, while *P. maritimus* rose from 0% to 4.54% (Figure 5F). This result suggested that certain *Psychrobacter* species exhibited greater resilience to HPP and could proliferate during refrigerated storage, potentially impacting seafood quality (Figure 5F). Overall, HPP at 400 MPa for 4 min was proven to be an effective intervention for reducing microbial populations in PC-EP. The treatment significantly suppressed spoilage and pathogenic bacteria, particularly *Aeromonas*, *Pseudomonas*, *Shewanella*, and *Vibrio*, thereby extending shelf life and enhancing seafood safety. However, the resilience of certain *Psychrobacter* species highlighted the need for additional potential preservation strategies.

Alpha diversity indices further confirmed these microbial shifts. In the control sample, the *observed species* and *Shannon* indices increased from 317.00 and 5.15 on day 0 to 399.00 and 5.32 on day 12, respectively, reflecting greater microbial diversity over time. However, in the 400 MPa-treated sample, these indices declined significantly. The *observed species* declined from 273.00 on day 0 to 176.00 on day 12, and *Shannon* decreased from 4.94 to 4.16. This suggests that HPP substantially reduced microbial diversity during refrigerated storage. These findings align well with those of Sameli et al. [39], who reported that extended refrigeration decreased bacterial diversity and abundance in shellfish since physiological activity was inactivated in some certain bacteria over time, thus altering the microbial community structure.

### 3.6. Volatile Organic Compounds of Precooked Baby Clam Edible Portion Subjected to HPP Under Selected Conditions

**Figure 6:**
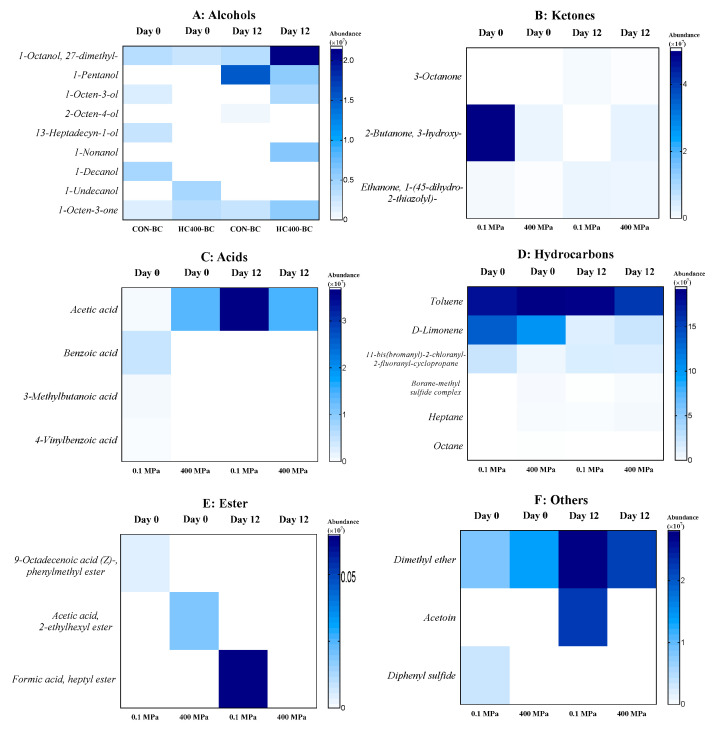
Heat map of volatile organic compounds of precooked baby clam edible portion without and with HPP treatment at 400 MPa on day 0 and day 12 of refrigerated storage.

The volatile organic compounds (VOCs) in the PC-EP of the control sample and HPP-treated sample (400 MPa for 4 min) on days 0 and 4 of refrigerated storage were determined (Figure 6). The major compounds examined included alcohols, ketones, acids, hydrocarbons, esters, and other compounds.

Alcohols significantly contribute to the sensory attributes of seafoods, imparting fresh and slightly sweet aromas. On day 0, both the control and HPP-treated samples exhibited relatively low alcohol levels (Figure 6A). On day 12, 1-Octanol and 2,7-dimethyl- increased notably in the 400 MPa-treated sample, suggesting better retention by HPP. Nonetheless, 1-pentanol was absent on day 0, but was detected on day 12, in which a higher concentration was found in the control than the HPP-treated sample, likely due to high microbial growth or oxidative processes in the former. Additionally, 1-Nonanol and 1-Octen-3-ol were detected in the HPP-treated sample on day 12 but were absent in the control, indicating that HPP might influence lipid oxidation pathways.

Ketones, formed through lipid oxidation and microbial metabolism, impact seafood flavor, but can lead to off-flavors when accumulated. 2-Butanone- 3-hydroxy- was initially high in the control but declined to undetectable levels on day 12 (Figure 6B). In contrast, it remained stable in the HPP-treated sample, suggesting that HPP retarded its degradation. Similarly, 1-Octen-3-one increased over time in both samples but was more prominent in the 400 MPa-treated sample, indicating a slower oxidation process under high pressure. Additionally, 3-Octanone appeared on day 12 in both samples, but was detected at significantly lower levels in the HPP-treated sample.

Acids are key spoilage indicators, as they result from microbial activity and biochemical degradation. Acetic acid, a marker of fermentation and microbial metabolism, increased significantly in the control sample (from 0.09 on day 0 to 3.71 on day 12) (Figure 6C). However, in the HPP-treated sample, this increase was much less pronounced, demonstrating the efficiency of HPP in suppressing microbial spoilage. Other acids, such as benzoic acid, 3-methylbutanoic acid, and 4-vinylbenzoic acid, were detected in the control sample on day 0 but disappeared on day 12, likely due to the microbial conversion of those compounds into secondary metabolites.

Hydrocarbons, which contribute to the aroma profile of seafoods, can also be part of lipid oxidation byproducts. Toluene remained relatively stable in the control but slightly decreased in the HPP-treated sample, suggesting that high pressure influenced the retention of this compound. D-Limonene, a citrus-scented hydrocarbon, declined in both samples, but a more noticeable change was found in the control (Figure 6D). This indicated better flavor retention in the HPP-treated sample. Additionally, heptane and the borane–methyl sulfide complex were present at higher levels in the 400 MPa-treated sample over time, highlighting the formation of distinct hydrocarbon profiles under high-pressure conditions.

During seafood spoilage, spoilage-associated bacteria such as *Pseudomonas*, *Shewanella*, *Enterobacteriaceae*, LAB, and *Aeromonas,* generate metabolic byproducts, including alcohols, ketones, aldehydes, organic acids, and esters [40]. This study demonstrates that HPP at 400 MPa effectively modified the VOC profile of PC-EP by delaying spoilage while preserving desirable flavor compounds. The reduced accumulation of acetic acid, formic acid, heptyl ester, and acetoin suggested that HPP inhibited microbial activity and extended the shelf life of PC-EP (Figure 6C,E,F). Additionally, variations in alcohol and hydrocarbon profiles indicated that HPP positively influenced lipid oxidation and enhanced flavor retention in PC-EP.

### 3.7. Acceptability of Precooked Baby Clam Edible Portion Subjected to HPP Under Selected Conditions

The sensory acceptability of the control (0.1 MPa) and HPP-treated samples (400 MPa) was evaluated before and after 12 days of refrigerated storage (Table 2). On day 0, the likeness scores for the control and 400 MPa-treated sample showed no difference (*p* > 0.05), indicating that HPP treatment at 400 MPa had no impact on the sensory acceptability of the samples. Notably, the 400 MPa-treated sample exhibited a firmer texture than the control, likely due to the compact alignment of myofibrillar proteins under high pressure. After 12 days of storage, the sensory likeness score of the 400 MPa sample remained stable, and there were no significant differences from the corresponding sample on day 0 (*p* > 0.05). The appearance, color, odor, texture, taste, and overall likeness scores on day 12 were 7.11, 7.00, 7.10, 7.00, 6.90, and 7.02, respectively. Generally, prolonged refrigerated storage of aquatic products brings about the formation of volatile nitrogenous basic compounds from protein decomposition and carbonyl compounds from protein oxidation, both of which negatively impact sensory attributes, particularly odor, flavor, and taste [41]. However, in this study, all likeness scores for all attributes remained above the minimum acceptable limit (score = 5). HPP thus contributed to texture preservation during refrigerated storage and helped reduce the quality deterioration of PC-EP [42].

## 4. Conclusions

High-pressure processing (HPP), particularly at high levels (400 or 600 MPa), could maintain the quality as indicated by the retarded increases in microbial load, total volatile basic nitrogen (TVB-N), trimethylamine nitrogen (TMA-N) contents, and pH. In addition, degradation of protein and formation of undesirable volatile compounds were also impeded when kept up to 12 days. The shelf life was extended to 12 days for the HPP-treated sample (400 MPa for 4 min). Throughout refrigerated storage, microbial diversity in the HPP-treated sample declined. On day 12, HPP treatment at 400 MPa effectively inhibited the growth of *Aeromonas* spp., *Shewanella* spp., and *Pseudomonas* spp., while *Carnobacterium* spp., *Psychrobacter* spp., and *Lactococcus* spp. became dominant. Therefore, HPP at 400 MPa significantly preserved the quality and safety of PC-EP by suppressing dominant spoilage bacteria. Overall, this study provides valuable insights into precooked baby clams, enhancing consumer awareness of their quality and safety for consumption.

## Figures and Tables

**Table 1:** Chemical properties of precooked baby clam edible portion subjected to HPP at different pressure levels during refrigerated storage.

Parameters	Samples	Day 0	Day 3	Day 6	Day 9	Day 12	Day 15
TVB-N content (mg N/100 g)	0.1 MPa	1.11 ± 0.14 ^aF^	2.13 ± 0.15 ^aE^	3.30 ± 0.09 ^aD^	5.46 ± 0.08 ^aC^	10.95 ± 0.12 ^aB^	13.06 ± 0.32 ^aA^
	200 MPa	0.91 ± 0.09 ^abF^	1.26 ± 0.04 ^aE^	2.37 ± 0.10 ^aD^	4.22 ± 0.08 ^aC^	5.34 ± 0.07 ^aB^	7.56 ± 0.05 ^bA^
	400 MPa	0.82 ± 0.01 ^bE^	0.94 ± 0.05 ^bE^	1.87 ± 0.04 ^bD^	2.79 ± 0.01 ^bC^	3.36 ± 0.05 ^bB^	4.88 ± 0.07 ^cA^
	600 MPa	0.79 ± 0.00 ^bE^	0.92 ± 0.01 ^bE^	1.85 ± 0.03 ^bD^	2.77 ± 0.04 ^bC^	3.35 ± 0.01 ^bB^	4.20 ± 0.09 ^dA^
TMA-N content (mg N/100 g)	0.1 MPa	0.33 ± 0.12 ^aF^	0.78 ± 0.01 ^aE^	1.13 ± 0.11 ^aD^	2.45 ± 0.07 ^aC^	5.46 ± 0.11 ^aB^	7.48 ± 0.21 ^aA^
	200 MPa	0.35 ± 0.06 ^aE^	0.57 ± 0.00 ^bDE^	0.75 ± 0.09 ^bD^	1.04 ± 0.08 ^bC^	3.49 ± 0.12 ^bB^	4.05 ± 0.13 ^bA^
	400 MPa	0.27 ± 0.03 ^aE^	0.43 ± 0.09 ^bDE^	0.64 ± 0.04 ^bD^	1.04 ± 0.00 ^bC^	1.74 ± 0.12 ^cB^	2.26 ± 0.10 ^cA^
	600 MPa	0.25 ± 0.05 ^aE^	0.40 ± 0.10 ^bDE^	0.62 ± 0.08 ^bD^	0.95 ± 0.05 ^bC^	1.64 ± 0.10 ^cB^	2.03 ± 0.09 ^dA^
pH	0.1 MPa	6.75 ± 0.01 ^aA^	6.62 ± 0.02 ^bB^	6.50 ± 0.03 ^cC^	6.20 ± 0.04 ^bD^	5.75 ± 0.03 ^cE^	5.39 ± 0.02 ^dF^
	200 MPa	6.76 ± 0.04 ^aA^	6.70 ± 0.01 ^aB^	6.60 ± 0.02 ^bC^	6.25 ± 0.02 ^bD^	5.85 ± 0.01 ^bE^	5.56 ± 0.03 ^cF^
	400 MPa	6.77 ± 0.02 ^aA^	6.75 ± 0.03 ^aAB^	6.70 ± 0.02 ^aB^	6.63 ± 0.02 ^aB^	6.56 ± 0.02 ^aB^	6.50 ± 0.01 ^bB^
	600 MPa	6.76 ± 0.03 ^aA^	6.74 ± 0.01 ^aAB^	6.70 ± 0.01 ^aB^	6.60 ± 0.01 ^aC^	6.61 ± 0.04 ^aC^	6.57 ± 0.01 ^aC^

Different uppercase superscripts in the same row denote significant difference (*p* < 0.05). Different lowercase superscripts in the same column within the same parameter denote significant difference (*p* < 0.05). The results are presented as means ± standard deviation (*n* = 3).

**Table 2:** Likeness score of precooked baby clam edible portion without and with HPP treatment at 400 MPa on day 0 and day 12 of refrigerated storage.

Attributes	Day 0		Day 12
	0.1 MPa	400 MPa	400 MPa
Appearance	7.45 ± 0.96 ^a^	7.43 ± 0.98 ^a^	7.11 ± 1.00 ^a^
Color	7.21 ± 0.96 ^a^	7.15 ± 0.94 ^a^	7.00 ± 0.93 ^a^
Smell	7.17 ± 0.85 ^a^	7.22 ± 0.91 ^a^	7.10 ± 0.87 ^a^
Texture	6.95 ± 0.73 ^a^	7.38 ± 0.64 ^a^	7.00 ± 0.85 ^a^
Taste	6.98 ± 0.84 ^a^	7.13 ± 0.82 ^a^	6.90 ± 0.85 ^a^
Overall	7.21 ± 0.91 ^a^	7.25 ± 0.82 ^a^	7.02 ± 0.89 ^a^

Different lowercase superscript letters in the same row denote significant differences (*p* < 0.05). The results are presented as means ± SD (*n* = 3).

## Data Availability

The original contributions presented in the study are included in the article; further inquiries can be directed to the corresponding author.
